# Monitoring Biogenic Amines: Comparative Assessment of Detection Methods for Key Market Marine and Freshwater Species

**DOI:** 10.1111/1750-3841.71083

**Published:** 2026-04-29

**Authors:** Lasse Petersen

**Affiliations:** ^1^ Bio and Food Technology, Faculty of Energy and Life Science Flensburg University of Applied Sciences Flensburg Schleswig‐Holstein Germany

## Abstract

Biogenic amines, primarily histamine, cadaverine, and putrescine, are important indicators of microbial spoilage and potential health risks. This review summarizes established and novel analytical methods for quantitatively determining biogenic amines in fish and fish products, along with their limits of detection in these complex matrices. Traditional chromatographic methods (HPLC/UPLC, GC, and capillary electrophoresis) with pre‐ or postcolumn derivatization offer the highest sensitivity, with detection limits down to the microgram range. Spectroscopic techniques (UV/Vis, fluorescence, NIR, Raman/SERS, and NMR) enable noninvasive, partially nondestructive, and inline measurements. Enzymatic, aptamer‐based, and molecular imprinting sensors, as well as immunoassay‐based methods (e.g., ELISA and immunosensor approaches), are advantageous for their speed, ease of use, and portability. Moreover, the review discusses the opportunities and limitations of these methods in terms of sensitivity, selectivity, and suitability for determining biogenic amines in the most market‐relevant species of marine and freshwater fish, while also providing a unique list of all marine and freshwater fish species that have already been examined for their biogenic amine content, thereby providing references for efficient and suitable detection methods in fish.

**Abbreviations**
AOX: amino oxidases
BSTFA: bis(trimethylsilyl)trifluoroacetamide
CE: capillary electrophoresis
DAO: diamine oxidase
EFSA: European Food Safety Authority
ELISA: enzyme‐linked immunosorbent assay
FDA: Food and Drug Administration
FLD: fluorescence detector
GC: gas chromatography
HPLC: high‐performance liquid chromatography
MAO: monoamine oxidase
MIP: molecular imprinted polymer
MS: mass spectrometry
NIR: near‐infrared
NMR: nuclear magnetic resonance
PCA: principal component analysis
PCR: polymerase chain reaction
PLS: partial least squares
SAM: self‐assembled monolayer
SELEX: Systematic Evolution of Ligands by eXponential enrichment
SERS: surface‐enhanced Raman spectroscopy
SPR: surface plasmon resonance
UV: ultraviolet

## 1 Introduction

In the seafood industry, freshness is a critical quality attribute of fish products relevant to consumer safety. Spoilage begins immediately after capture, leading to sensory deterioration such as unpleasant odors and flavors and textural changes. Spoilage results in economic losses and potential health risks. In this process, biogenic amines play a crucial role in food as indicators of freshness and spoilage because of their involvement in physiological functions in organisms and toxicity (Gram and Huss 1996). Biogenic amines, such as cadaverine, putrescine, and histamine, are formed as decarboxylation products of amino acids caused by microbes. Their concentration is correlated with microbial spoilage in fish. Cadaverine (derived from l‐lysine) and putrescine (derived from l‐arginine) accumulate during storage, reflecting the degree of bacterial proliferation (Middlebrooks et al. 1988). Especially in food, they can potentially harm human health, as a high intake of biogenic amines is associated with health risks. Most biogenic amines have inhibitory, allergenic, or toxic potential. Permissible concentrations of typical biogenic amines are regulated by the EU Regulation 2073/2005 (European Union 2005). Therefore, quantitative monitoring of these amines can be used to indicate freshness and ensure public health (European Union 2005).

Accordingly, several methods have been developed for detecting biogenic amines. Stringent safety regulations and rising quality expectations have created a growing demand for rapid, reliable, and cost‐effective analytical methods. Traditional approaches rely on chromatographic techniques, such as high‐performance liquid chromatography (HPLC) and gas chromatography (GC), with pre‐ or postcolumn derivatization to achieve high sensitivity and selectivity (Munir et al. 2020). Recently, enzymatic, optical, or electrochemical biosensors and immunoassays have shown promise for rapid on‐site or at‐line screening (Munir et al. 2020). Additionally, vibrational spectroscopies, such as near‐infrared (NIR) and Raman, offer the potential for nondestructive, real‐time assessments in processing lines (Z. Zhang et al. 2022).

Several reviews on the topic of biogenic amines in food have already been published, focusing rather on the different factors influencing the formation of biogenic amines (Gardini et al. 2016; Wójcik et al. 2021), biogenic amines in specific groups of food such as fish and fish products (Al Bulushi et al. 2009; Prester 2011; Visciano et al. 2020), or the different detection methods (Önal et al. 2013; Papageorgiou et al. 2018; Ahangari et al. 2021; Givanoudi et al. 2023; Tırıs et al. 2023; Z. Chen et al. 2024).

Despite that, there is a gap in the literature regarding the direct comparison of detection methods for biogenic amines, especially regarding their analytical performance in complex matrices such as fish. Therefore, this review will provide an overview of established and emerging methods for detecting biogenic amines in fish and evaluate their analytical performance and practicability. Moreover, the review will provide a unique overview of all already examined marine and freshwater fish species, including those from more local markets, regarding their biogenic amine content, enabling a straightforward method choice and data analysis on a species‐specific level.

## 2 Biogenic Amines in Fish and Fish Products

### 2.1 Factors Influencing Biosynthesis and Accumulation

**FIGURE 1:**
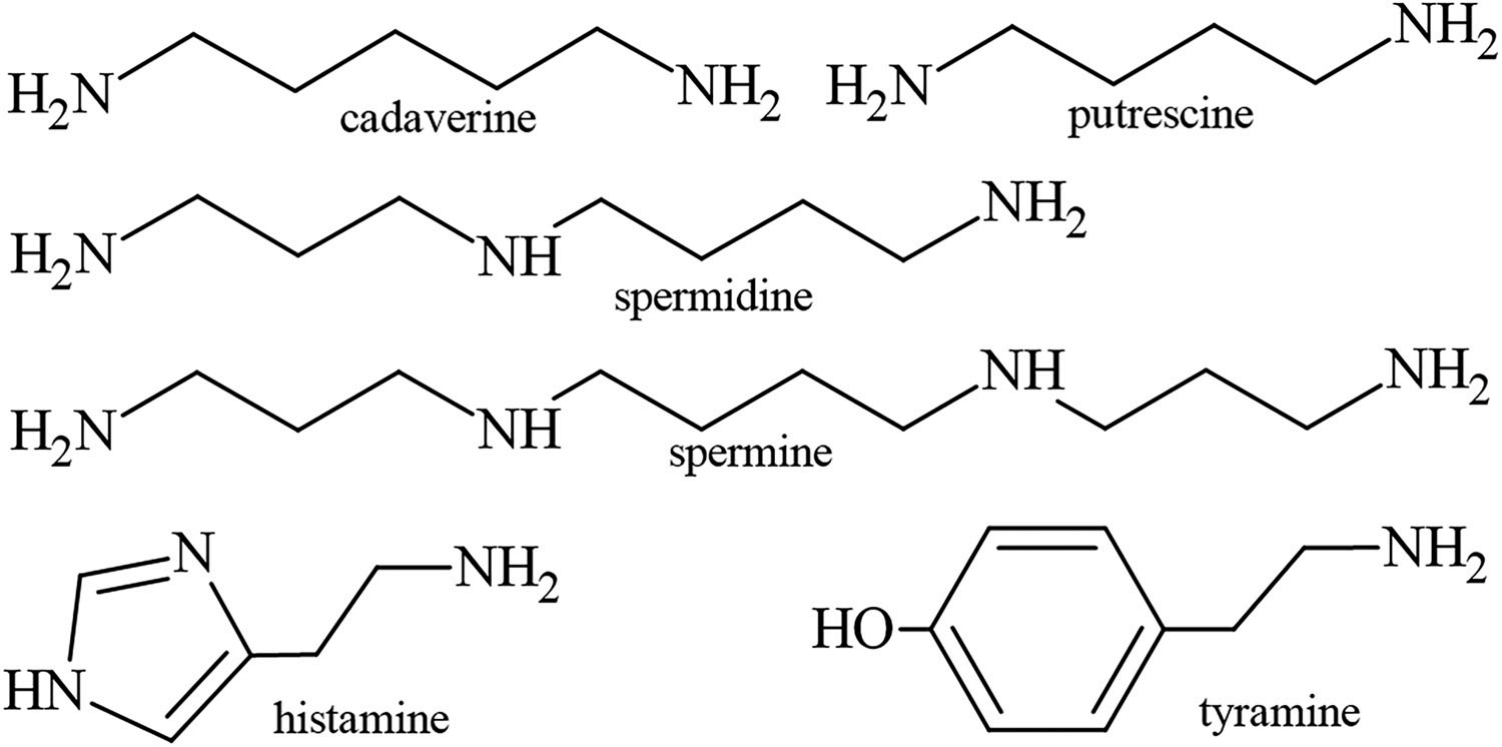
Chemical structures of primary biogenic amines.

Biogenic amines are organic compounds that contain nitrogen and are formed mainly by the decarboxylation of amino acids. Biogenic amines are commonly found in all living organisms, including humans and animals. Also, they appear as relevant spoilage products in protein and amino acid degradation (Silla Santos 1996). The most common and noteworthy spoilage‐associated biogenic amines in fish are histamine, cadaverine, putrescine, and tyramine. Also, spermine and spermidine, which are formed from putrescine, can be found in fish (Zarei et al. 2011) (Figure 1).

Biogenic amines are categorized by the quantity of amino groups (monoamines, diamines, and polyamines) (Biji et al. 2016) or their chemical composition (aromatic, aliphatic, and heterocyclic biogenic amines) (Silla Santos 1996). Furthermore, biogenic amines can be classified as endogenous or exogenous based on their origin. Endogenous biogenic amines have biological functions and are synthesized directly by organisms, whereas exogenous biogenic amines are obtained through dietary intake (Ahangari et al. 2021).

Amino acid decarboxylation products are formed during normal metabolic activities in living cells. Decarboxylase enzymes are found in animal and plant cells, as well as in microorganisms. However, bacteria generally possess high decarboxylase activities (Shalaby 1996).

**FIGURE 2:**
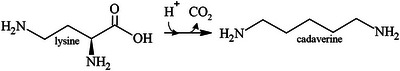
Formation of cadaverine by lysine decarboxylase catalysis of lysine.

In general, amino acid decarboxylases are enzymes that catalyze the formation of biogenic amines by the removal of the carboxyl group (–COO) (Figure 2). Other mechanisms of amine formation include amination and transamination of aldehydes and ketones (Rehner and Daniel 2010).

Amino acids, as essential building blocks of peptides and proteins, are ubiquitously present in all living organisms, making them a readily available source for the synthesis of biogenic amines (Rehner and Daniel 2010). Especially after the death of a living organism, which typically results in the release of proteins and autolytic protein degradation, amino acids are liberated. Upon further biochemical transformation, these free amino acids can be converted into biogenic amines through enzymatic processes. The accumulation of these amines is often associated with the onset of putrefaction, influencing the preservation and decomposition processes in both natural and controlled environments (Rehner and Daniel 2010).

The formation and accumulation of biogenic amines require certain fundamental factors: first, the presence of the corresponding amino acids; second, the presence of microorganisms that can exhibit decarboxylase activity; and third, the environmental conditions, which must allow the growth of microorganisms and their enzyme activity (Biji et al. 2016). Deteriorating microorganisms are already in the raw material as part of the natural microflora. They can also result from contamination during catch and slaughter (Huis in ’t Veld 1996). Numerous environmental conditions and factors can influence the formation of biogenic amines, starting from the raw material, which has a crucial influence on the formation of biogenic amines based on its respective composition, especially protein content and the presence of free amino acids (Ruiz‐Capillas and Herrero 2019). Food, especially meat and fish with a high protein content, fosters microbial growth and subsequent formation of biogenic amines. Many biogenic amines, such as spermidine, spermine, or putrescine, already occur naturally in meat and muscle due to endogenous biosynthesis in, for example, proliferating cells and their involvement in cell division or regeneration (Halász et al. 1994; Silla Santos 1996).

Various microorganisms show decarboxylase activities and play a crucial role in forming biogenic amines in food and fish. Gram‐negative Enterobacteriaceae are one of the most important microorganisms that can form biogenic amines, including *Enterobacter* spp., *Escherichia coli*, and *Klebsiella* spp. *Morganella morganii* is known as one of the strongest histamine producers in fish (Oktariani et al. 2022; Ucar and Ozogul 2024). Besides these, *Photobacterium* spp., *Aeromonas* spp., and gram‐positive lactic acid bacteria can contribute to the formation of biogenic amines (Oktariani et al. 2022; Ucar and Ozogul 2024). Specifically, *Pseudomonas* spp. are responsible for forming cadaverine and putrescine (Koutsoumanis et al. 1999; Ucar and Ozogul 2024).

Water activity, pH, and temperature of the raw material are additional environmental factors contributing to the formation of biogenic amines. The higher the water activity and temperature, the more likely biogenic amines are to be formed and accumulated. Therefore, handling the raw material and reducing spoilage microorganisms are the best ways to reduce biogenic amine formation (Gardini et al. 2016; Ucar and Ozogul 2024).

### 2.2 Toxicological and Sensory Effects

Endogenous biogenic amines contribute to various functions in the human body, including neurotransmission and hormonal regulation. Such biogenic amines also serve as precursors for nucleic acid and protein synthesis and contribute to important physiological functions in animals (Halász et al. 1994; Ahangari et al. 2021). When consumed in low doses, exogenous biogenic amines generally do not significantly impact human health. The human body has detoxification mechanisms, which involve the amine oxidase enzymes monoamine oxidase (MAO), diamine oxidase (DAO), and polyamine oxidase. These activities mainly occur in the small intestine, so only a small amount of the ingested biogenic amines can enter the bloodstream (Fogel et al. 2007; Ruiz‐Capillas and Herrero 2019; Kettner et al. 2022).

Nevertheless, several factors can contribute to toxicological issues, even in a healthy human body. In particular, the excessive consumption of spoiled or fermented foods increases the levels of biogenic amines and can result in health problems (EFSA 2011; Ruiz‐Capillas and Herrero 2019). Another contributing factor is the intake of inhibitors of amino oxidases (AOX), which can completely block their activity. Examples of such inhibitors include MAO and DAO inhibitor antidepressants (Mah et al. 2019; Ruiz‐Capillas and Herrero 2019).

The most health‐threatening biogenic amine is histamine (Ucar and Ozogul 2024). A small intake of 10–50 mg can cause an intolerance reaction, which occurs in symptoms such as a feeling of heat, itching of the skin, and hypersalivation. Vomiting, headache, circulatory symptoms, abdominal pain, and blood pressure drop can be caused by an intake of 100–1000 mg of histamine (Ruiz‐Capillas and Herrero 2019; Kettner et al. 2022; Ucar and Ozogul 2024). These poisoning symptoms occur within a few minutes to a few hours after eating food (Matissek 2020). The most common source of histamine poisoning is fish from the Scombridae family, which includes tuna and mackerel; therefore, the histamine intoxication is also called scombroid fish poisoning (Ruiz‐Capillas and Herrero 2019; Tabanelli 2020). Although histamine can also occur in fresh fish, spoiled fish can contain much higher histamine concentrations ranging from 2000 to 5000 mg/kg (Matissek 2020).

Despite their toxic properties, most other biogenic amines, such as cadaverine, enhance the toxic reactions of histamine rather than having their own toxicologic effects due to their high efficacious doses (Ucar and Ozogul 2024). Cadaverine, for example, enhances histamine transport across the intestinal wall and reduces histamine metabolism by disrupting intestinal absorption of histamine (Schirone et al. 2022; Ucar and Ozogul 2024). Additional concerns were raised as cadaverine and putrescine can lead to the formation of carcinogenic nitrosamines (Schirone et al. 2022).

Biogenic amines can affect the sensory perception of fish and their changes during storage. Sensory odor, taste, and texture changes correlate with microbial changes and can be recognized during the accumulation of different biogenic amines in food (Hu et al. 2012; Biesuz and Magnaghi 2021). Histamine can lead to the development of sour and metallic notes of odor and taste. Putrescine is known for its strong, putrid, rancid, or rotting smell. The taste is sometimes described as sharp or slightly sweetish. The appearance of cadaverine can lead to a fecal, sweaty, or moldy smell and can impart a bitter or slightly astringent flavor. Tyramine is also described as a bitter and astringent flavor contributing to the sharpness of degrading fish flesh (Prester 2011; Alizadeh et al. 2025). High biogenic amine levels correlate but are not causative with texture changes, which are based on microbial proteolysis and lead to soft, mushy, or slimy fish flesh. Also, a loss of firmness and increased drip loss can be recognized (Chytiri et al. 2004; Visciano et al. 2020).

### 2.3 Regulatory Aspects

Because biogenic amines can cause a profound impact on the overall food quality and safety, the Food and Drug Administration (FDA) in the United States and the European Food Safety Authority (EFSA) have implemented regulations for certain biogenic amines in fish. The most regulated biogenic amine is histamine, with respect to its health effects.

The FDA states that histamine above a 50 mg/kg level in scombroid fish must be considered unsafe (FDA 2022). The EU has laid down binding microbiological criteria for fishery products in EU Regulation 2073/2005. This regulation includes the regulation of histamine in scombroid fishes to be less than 100 mg/kg (European Union 2005).

Furthermore, different studies suggested possible guidelines for the histamine content of fish (Table 1).

**TABLE 1:** Possible guidelines for histamine content of fish.

Content (mg/kg)	Diagnosis	References
<50	Safe for consumption	Brink et al. 1990; Mah et al. 2019
50–200	Possibly toxic	
200–1000	Probably toxic	
>1000	Toxic and unsafe for human consumption	

There are currently no regulatory limits for other biogenic amines in fish, but several authors and institutions have proposed maximum tolerable levels, for example, AGES in Austria. For putrescine, a maximum tolerable level of 170 mg/kg was proposed for fish; for cadaverine in fish, the tolerable level is 510 mg/kg (Österreichische Agentur für Gesundheit und Ernährungssicherheit GmbH n.d). The EFSA, however, has proposed a safe threshold of 600 mg of tyramine per meal for healthy adults as part of a risk assessment. Significantly lower tolerance levels apply to individuals with specific sensitivities, such as those undergoing MAO inhibitor therapy (EFSA 2011). Another suggestion is Maurer's so‐called biogenic amine index (BAI), which suggests the total amount of the biogenic amines putrescine, cadaverine, histamine, and tyramine in the food sample as a marker of fish freshness. An amount above 150 mg/kg is considered not fresh and clearly spoiled (Ruiz‐Capillas and Herrero 2019).

### 2.4 Freshness Evaluation Methods

In the food industry, the most common methods for evaluating food freshness are still microbiological methods and sensory evaluation. While microbiological methods are time‐consuming, sensorial evaluation is subjective due to differences in test subjects’ perceptions. On the other hand, sensorial evaluation is a fast, easy‐to‐apply method that does not require any specialized instrumental prerequisites beyond standardized training of the sensory panel. To overcome the limitations of microbiological and sensory methods, there has been an increasing focus on chemical analysis approaches for determining freshness. These approaches quantitatively measure characteristic spoilage markers, such as volatile organic compounds, biogenic amines, and lipid oxidation products, providing objective and reproducible results independent of individual taste or smell impressions. Modern methods such as gas chromatography–mass spectrometry (GC–MS), HPLC, and NIR spectroscopy can also detect even the smallest concentrations quickly and automatically, often before any visible or noticeable loss of freshness occurs. In some cases, they can also be integrated into production processes online. This makes chemical methods a valuable addition to traditional food freshness evaluation methods.

## 3 Sample Extraction and Derivatization

Special requirements are derived from the complex sample matrix, which has potentially interfering compounds and mostly low concentrations of biogenic amines, to determine biogenic amines in food samples. Extraction, concentration, and derivation of the samples are needed. Various approaches and pretreatment technologies exist depending on the different sample matrices and their specificities. The choice of an appropriate sample pretreatment method is crucial for the analysis's accuracy, sensitivity, selectivity, and speed. Options are solid‐phase extraction, liquid–liquid extraction, or microextraction. The goal of these extraction processes is to remove potential interfering compounds and the concentration of the analytes (Sagratini et al. 2012; Huang et al. 2016; Q. Chang et al. 2019; Cao et al. 2019; Donthuan et al. 2014; Parchami et al. 2017; Ochi 2019; Guo et al. 2022).

The extraction of biogenic amines is commonly done with different acids such as trichloroacetic acid (TCA), hypochlorous acid (HClO), perchloric acid (HClO4), or hydrochloric acid (HCl) (Sagratini et al. 2012; Q. Chang et al. 2019; Ochi 2019; Plakidi et al. 2020; X. Zhang et al. 2021). The sample is first homogenized with the extraction solvent using a rotor–stator disperser, followed by centrifugation. The resulting supernatant, containing the biogenic amines from the sample, is then used for derivatization.

Derivatization is required in most chromatographic and spectroscopic detection methods because biogenic amines exhibit poor fluorescence. Common reagents for the derivatization of biogenic amines include *O*‐phthalaldehyde (OPA), which forms fluorescent isoindole derivatives in the presence of either mercaptoethanol or *N*‐acetylcysteine (NAC); dansyl chloride, which is a fluorescent marker that yields stable products upon prolonged incubation; or FMOC‐Cl (9‐fluorenylmethoxycarbonyl chloride), which is used to produce ultraviolet (UV)‐detectable carbamates. Derivatization methods differ in stability and reaction patterns; for example, OPA reacts only with primary amines and produces unstable derivatives. Dansyl chloride and dabsyl chloride stably label primary and secondary amines (dansyl: fluorescent/UV‐active; dabsyl: visibly colored) (Antoine et al. 1999; Bomke et al. 2009; Male and Luong 2001; Tahmouzi et al. 2011; Sagratini et al. 2012; Donthuan et al. 2014; Herrero et al. 2016; Cao et al. 2019).

Regarding the missing volatility, which is necessary for biogenic amine detection by GC, possible pretreatments are silylation with BSTFA (bis(trimethylsilyl)trifluoroacetamide), acylation with heptafluorobutyric anhydride (HFBA), trifluoroacetylation, or the combination with alkylation to form stable esters or carbamates (Munir et al. 2017; Petrarca et al. 2017).

## 4 Detection Methods

Traditional approaches mainly rely on chromatographic techniques hyphenated with the respective detection methods. Recently, the potential for nondestructive and real‐time spectroscopic detection methods was shown and developed, accompanied by immunoassays, promising for on‐site and at‐line screening approaches.

### 4.1 Chromatographic Methods

Chromatographic methods are the most commonly used methods for detecting biogenic amines. Due to hyphenation with MS and the pre‐ or postcolumn derivatization of biogenic amines, these methods exhibit high sensitivity and selectivity.

#### 4.1.1 Liquid Chromatography

HPLC and, nowadays, ultra‐performance liquid chromatography (UPLC) are applied in the majority of the described approaches, especially using reversed‐phase separation on C18 columns (Latorre‐Moratalla et al. 2009; G. Li et al. 2014; Plakidi et al. 2020).

Pre‐ or postcolumn derivatization is performed; precolumn derivatization improves the separation in reversed‐phase columns by reducing the polarity of the amines. Derivatives can be detected using coupled mass spectrometers (MS), fluorescence detectors (FLDs), or UV/Vis detectors.

HPLC–FLD or HPLC–MS/MS ensures high sensitivity in the low nanogram range. While the methods are exact, they are time‐consuming and require specialized laboratory equipment (Antoine et al. 1999; Bomke et al. 2009; Sagratini et al. 2012; Cao et al. 2019; Kočar et al. 2021; Kosma and Badeka 2021; T. Li et al. 2022).

#### 4.1.2 Capillary Electrophoresis (CE)

CE is the second most applied method for determining biogenic amines in food. Despite its lower sensitivity, CE offers several advantages, such as a simple, fast, and reliable analysis of large sample quantities and low reagent consumption, making it cost‐effective.

The sensitivity of CE can be increased using various approaches, such as offline or online prederivatization. Additional approaches include the use of specialized buffers without derivatization or ready‐to‐use kits. For the detection of the derivatives, indirect and electrochemical detection, as well as UV, photometric, and MS coupling, are used (Su et al. 2000; Male and Luong 2001; Cinquina et al. 2004; Chiu et al. 2006; Rossano et al. 2006).

#### 4.1.3 GC

Only a few studies employed GC–MS and GC–MS/MS to detect biogenic amines in food samples. Due to the missing volatility of biogenic amines, a pretreatment of the samples is obligatory. Depending on the detection and determination method, different derivatization reagents, namely, BSTFA and HFB, can be used. GC–MS methods have proven exact identification in complex sample matrices, but high equipment costs and sample preparation limit their use in routine analyses (Huang et al. 2016; Munir et al. 2017; Munir et al. 2021).

### 4.2 Spectroscopic Methods

Spectroscopic methods have become well‐established as fast, highly sensitive, and selective tools for determining biogenic amines. Spectroscopic methods can detect concentrations down to the picogram range through targeted derivatization, with minimal sample preparation. Their automation capabilities and short measurement times make them ideal for high‐throughput analyses, and optional coupling to HPLC or CE increases selectivity and signal assignment. Furthermore, many spectroscopic methods are less invasive than chromatographic detection methods. Together, these methods enable the efficient and reliable analysis of biogenic amines in complex matrices.

#### 4.2.1 UV/Vis Spectroscopy

To detect biogenic amines, the amines are typically derivatized prior to measurement. The derivatives absorb strongly in the UV/Vis range and can be quantified. Furthermore, the production of different chemical metabolites during the spoilage process alters the absorption spectra and intensity, which can be used to determine the sample's age.

Often, this method is used alongside a preliminary liquid chromatography method for improved selectivity. It is simple, inexpensive, and robust. However, it only achieves detection limits in the micromolar to millimolar range and is more susceptible to matrix interference and, therefore, is often used for fast routine testing.

Matrix interferences can occur due to the complexity of the sample matrix. The leading causes are proteins, peptides, amino acids, lipids, sugars, and color pigments, whose strong absorbance bands can overlap the absorbance bands of biogenic amines. Derivatization can also lead to the formation of UV‐active byproducts or reaction residues that can overlap the absorbance bands of biogenic amines (Cinquina et al. 2004; Pinto et al. 2016; Plakidi et al. 2020).

#### 4.2.2 Fluorescence Spectroscopy

Fluorescence spectroscopy is a highly sensitive method for the detection of biogenic amines. As with UV/Vis spectroscopy, derivatization of the biogenic amines is required before analysis because they lack fluorescent properties. For better selectivity, fluorescence spectroscopy is usually coupled with a prior HPLC or CE.

In contrast to UV/Vis spectroscopy, the advantage is the much higher sensitivity down to nanomolar or micromolar concentrations in linear ranges, with a lower demand for sample amount. Recovery rates in model matrices are greater than 90%. Influencing factors, such as matrix interferences from samples, are compensated for by suitable calibration methods, such as matrix‐matching or standard addition. The downside is that it requires more complex sample and instrument preparation, making it a time‐consuming and expensive method.

Therefore, fluorescence spectroscopy is more commonly used for precise trace analysis in fish. Furthermore, in combination with other methods, a possible use is the nuanced characterization of different fish species or storage conditions (Latorre‐Moratalla et al. 2009; Tahmouzi et al. 2011; Zotou and Notou 2012; G. Li et al. 2014; Plakidi et al. 2020).

#### 4.2.3 NIR Spectroscopy

NIR spectroscopy is a nondestructive, objective, and cost‐effective online detection method for determining fish freshness. Another advantage is that sample preparation is minimal or not required at all.

Multivariate calibration of the NIR method is necessary for detecting biogenic amines. For example, reference measurements of fish samples of varying degrees of spoilage must be taken using HPLC to quantify biogenic amine content. These results can be used as a reference for different sample matrices. The recorded NIR spectra must then be set with respect to the reference sample. Due to complex sample matrices, high error rates can occur, and the differentiation between different biogenic amines becomes more difficult. Therefore, accurate and robust chemometric modeling, for example, principal component analysis (PCA) or partial least squares (PLS) regression, is necessary.

Once a robust reference database is established, the NIR offers a fast determination method for detecting biogenic amines with detection limits in the milligrams per kilogram range (Qu et al. 2015; Shim and Jeong 2019; Zhong et al. 2024).

#### 4.2.4 Raman Spectroscopy

The Raman spectroscopy, especially with surface reinforcement (surface‐enhanced Raman spectroscopy [SERS]), is another fast, nondestructive detection method for biogenic amines. The principle of this detection method is the Raman effect, which describes the scattering of monochromatic light (a laser) by molecules and provides spectral fingerprints of their molecular vibrations.

The advantages of this method include reduced sampling effort, the need for fewer reaction reagents during sample preparation, and its label‐free nature. Due to characteristic Raman bands of the amines, the measurement is molecule specific.

Low biogenic amine contents of the sample can lead to problems regarding the low Raman scattering probability of the samples, whereby the detection limits are high. Furthermore, matrix interferences can occur due to sample components such as proteins and colorants, which can cause signal suppression or overlapping Raman bands, leading to difficulties in quantifying the biogenic amines. Data analysis often involves multivariate statistics (e.g., PLS regression, PCA) and is therefore rather complex. Another disadvantage is the high acquisition and operating costs associated with the price of high‐quality Raman systems and SERS substrates (Cheng et al. 2013).

To address the problem of high detection limits, SERS is often used instead of Raman spectroscopy, especially as part of biosensors. Nanostructured metal surfaces (Ag, Au) can amplify Raman signals by factors of 10^4^ to 10^8^ and lower detection limits to the nanomolar to micromolar range (Bingquan et al. 2017; Janči et al. 2017; Xie et al. 2017).

#### 4.2.5 Nuclear Magnetic Resonance (NMR)

NMR spectroscopy is a spectroscopic method for investigating molecules via the magnetic properties of their nuclear spins. Therefore, a strong magnetic field is needed. The advantages of this detection method include its lower destructiveness: only a small sample is needed, no sample derivatization is required, and minimal sample preparation is required. Moreover, NMR is a highly sensitive detection method that enables multicomponent analysis of multiple amines and other metabolites in a single spectrum. Due to the similar structures of biogenic amine metabolites, quantitative determination of BAs is difficult because of overlapping signals. Similarly, matrix effects can occur in complex sample matrices, leading to strongly overlapping background signals (Cheng et al. 2013; Shumilina et al. 2015). Combining with other methods, such as liquid chromatography, can enhance sensitivity. As with Raman spectroscopy, high acquisition and operating costs can be seen as disadvantages of this detection method (Cheng et al. 2013).

It is more commonly used for quality evaluation, including fat content and distribution, water holding capacity, collagen content, pH, and metabolites (Cheng et al. 2013). Only a few studies have investigated the potential of the NMR for detecting and quantifying biogenic amines (Cheng et al. 2013; Shumilina et al. 2015; Shumilina et al. 2016). Therefore, further studies are needed to investigate the practicability and reliability of qualitative and quantitative detection of biogenic amines.

### 4.3 Biosensors

Other reviews and studies have not clearly categorized bio‐ and chemosensors for detecting biogenic amines by their biorecognition elements and transducing methods.

Therefore, this review categorizes biosensors according to their biorecognition element, immobilization method, and the commonly used transducing method.

**FIGURE 3:**
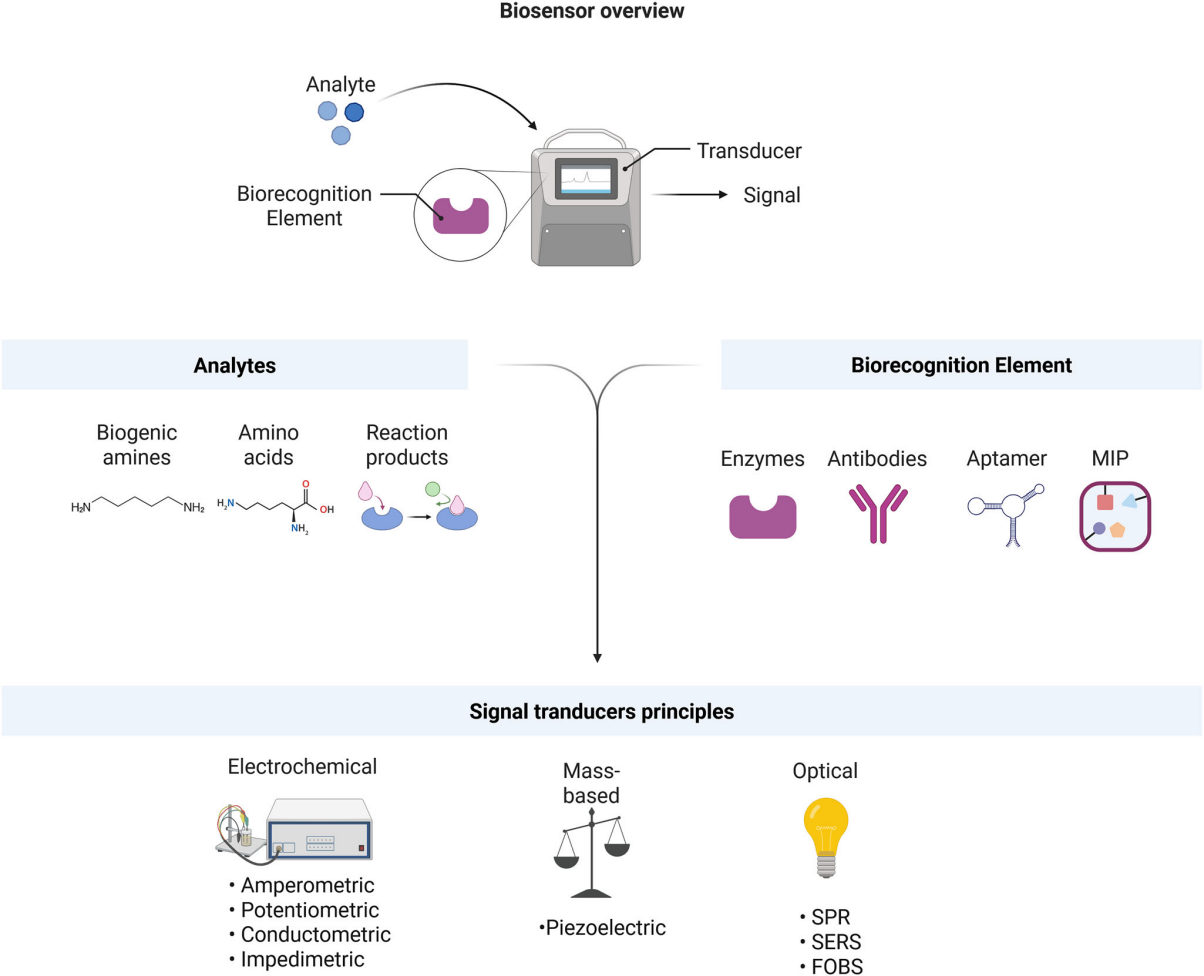
Schematic diagram of biosensor designs.

In general, a typical biosensor consists of a biorecognition element, which serves as a selective binding option for the biogenic amine, immobilized on an electrode, and a transducing method to detect the electrical or optical signal of the analytes or the reaction products and thereby the presence or amount of biogenic amines (Figure 3) (Z. Chen et al. 2024; Vasconcelos et al. 2021).

#### 4.3.1 Biorecognition Elements

##### 4.3.1.1 Biological

Most commonly used biological biorecognition elements are enzymes. These enzymatic biosensors use different AOX to detect biogenic amines. MAO, DAO, or polyamine oxidase can be used depending on the intended usage. The combination of different AOX can determine the general biogenic amine content. Specific oxidases or enzymes must be used to determine a specific biogenic amine (Lange and Wittmann 2002; Pérez et al. 2013; Apetrei and Apetrei 2016; Trevisani et al. 2019). Antibodies can also be used to detect the desired biogenic amine precisely.

Enzymes are difficult to extract, isolate, and purify and are therefore quite expensive. Often, mediators and nanomaterials are used to enhance electrochemical performance and catalytic efficiency, thereby improving sensitivity and reducing enzyme degradation (Lange and Wittmann 2002; Pérez et al. 2013; Apetrei and Apetrei 2016; Trevisani et al. 2019).

Antibodies are the second possible biological biorecognition element. Like enzymes, antibodies are expensive because of their manufacturing process, which typically involves immunizing animal cells. On the other hand, antibodies are specific, biogenic amines bind with high affinity, and detection limits in the nanogram range can be achieved (Dong et al. 2017; Shkodra et al. 2020; Peng et al. 2022).

##### 4.3.1.2 Aptamer

Aptamers are short single‐stranded DNA and RNA sequences or nucleic acid analogs that act as ligands for specific molecular targets, such as biogenic amines. They are produced with the so‐called Systematic Evolution of Ligands by eXponential enrichment (SELEX) from random oligonucleotides. The production steps follow a similar procedure to antibody production. For RNA aptamers, (i) denaturation of the oligonucleotides is followed by (ii) binding to the target analyte, (iii) elution of the bound aptamers, (iv) polymerase chain reaction (PCR) amplification, and (v) final retrieval of the aptamers. RNA aptamers are produced similarly, with an additional reverse transcription step prior to PCR and an in vitro transcription step after amplification. Nucleotide aptamers adopt a three‐dimensional conformation, and further tertiary folding occurs upon target binding (Dwidar and Yokobayashi 2019; Lerga et al. 2019; Ho et al. 2020; Duan et al. 2022; C. Li et al. 2023).

Aptamers, as biorecognition elements, offer crucial advantages over enzymes and antibodies, including greater stability, easier modification, and potential reusability. Due to in vitro production, aptamers are also less expensive.

Regarding disadvantages, aptamer affinity problems can occur, so biogenic amine analogs and structurally related amines may also bind.

The limit of detection, depending on the transducing method used, ranges from micromolar to nanomolar (Dwidar and Yokobayashi 2019; Lerga et al. 2020).

##### 4.3.1.3 Molecular Imprinted Polymers (MIPs)

MIPs are synthetic polymers formed of a three‐dimensional matrix with micro‐ or nanocavities specific for the target molecule. A copolymerization of functional monomers in the presence of the template and cross‐linkers for stability occurs due to the monomers’ self‐assembly driven by weak electrostatic interactions. After template extraction, microcavities within the polymer that are complementary to the targets are formed. These act as recognition sites for the targets in a reversible manner (Mattsson et al. 2018; Saylan et al. 2019; Munir, Rahmawati, et al. 2023).

The advantages of MIPs include long‐term storage and repeated use after extracting the captured molecules. MIPs are usually resilient to many organic solvents and nonphysiological conditions. Moreover, MIPs, such as antibodies, are highly specific and are already successfully synthesized to detect histamine, tyramine, tryptamine, putrescine, and dopamine. Ranges from micrograms to milligrams can be achieved as the limit of detection (Horemans et al. 2010; Song et al. 2010; Huang et al. 2011; Meng et al. 2014; Ying et al. 2018; Ying et al. 2020).

Due to the sample's pH, there can be problems with the target‐binding affinity because of the protonation states of biogenic amines, which can affect noncovalent binding to the MIP (Bongaers et al. 2010; Trikka et al. 2012).

#### 4.3.2 Immobilization Methods

Different immobilization methods are suitable for detachment from the sensor or electrode, depending on the intended biorecognition element. The immobilization supports the biorecognition element or is part of the transduction mechanism. Therefore, the activity of the biorecognition element must remain on the transducer.

##### 4.3.2.1 Adsorption

Often, microorganism cells or enzymes are incubated with an electrode or an immobilization matrix for attachment using adsorption. Adsorption is driven by van der Waals forces, hydrogen bonding, electrostatic interactions, or hydrophobic interactions between microorganisms or enzymes and the electrode or the immobilization matrix (Wiseman and Frank 2012; Rashid and Yusof 2017; Liu et al. 2018).

##### 4.3.2.2 Self‐Assembled Monolayer (SAM)

SAMs are thin, nanostructured films that form aggregates through the chemisorption of organic molecules onto the electrode surface. The advantage of this immobilization method is the ability to assemble individual molecules with different terminal groups onto a surface by controlling temperature and reaction conditions. The adsorbate interacts and binds to the substrate surface by immersing the substrate in an adsorbate dilution. As a result, a monomolecular film forms between the adsorbents (Chaki and Vijayamohanan 2002; Wu et al. 2019).

##### 4.3.2.3 Covalent Bindings

Covalent binding is the most potent enzyme‐binding method, providing high surface coverage when enzymes are used as biorecognition elements. Therefore, the enzyme is linked to thiols or amines during synthesis, which serve as functional groups and can bind to the electrode surface or to proteins, or to specific functional groups applied to the electrode surface in advance (Wong et al. 2009; Rashid and Yusof 2017; Sneha et al. 2019).

##### 4.3.2.4 Entrapment

Entrapment is an immobilization method in which the enzyme is physically confined within a polymer, thereby enhancing its mechanical stability. Furthermore, enzyme leaching and enzyme denaturation can be reduced. Therefore, nanostructured supports, such as electrospun nanofibers or pristine materials, for example, biopolymers or hydrogels, are designed to provide an optimal physicochemical environment for the enzyme. These nanostructures build porous networks or matrices through which the substrate passes. Analytes and enzymatic reaction products can diffuse (Datta et al. 2013; Sattar et al. 2018).

##### 4.3.2.5 Cross‐Linking

Enzymes can be cross‐linked via intermolecular reactions, such as covalent bonds, onto support matrices. This immobilization can be improved by using cross‐linked enzyme aggregates, cross‐linked enzyme crystals, or a combination of the two (Sneha et al. 2019; Thangaraj and Solomon 2019).

#### 4.3.3 Transducing Methods

##### 4.3.3.1 Electrochemical

Electrochemical transducing methods are based on chemically modified electrodes, yielding various sensor types depending on the employed modification approaches. Amperometric sensors detect the electric current through an electrode when an electric potential is applied (Keow et al. 2007; Pérez et al. 2013; Henao‐Escobar et al. 2016). An alternative approach is to utilize conductometric methods. The conductivity of a sample solution between two electrodes is determined by sensors of this type, thereby obviating the necessity for a reference electrode. These sensors are often utilized to detect putrescine, cadaverine, and histamine (Xing et al. 2012; Adley and Ryan 2015; Y. Li et al. 2017). Conversely, an impedimetric sensor can detect changes in the impedance of an electrochemical cell resulting from redox biochemical reactions as a function of frequency when biogenic amines react with the biorecognition element. These sensor types are primarily tested for histamine detection (Horemans et al. 2010; Bongaers et al. 2010; Peeters et al. 2013).

Furthermore, electrodes can be modified to detect changes in their electric potential upon contact with the analyte, namely, a biogenic amine. This particular sensor is designated as a potentiometric sensor. To detect, an indicator and a reference electrode are required. Histamine is predominantly evaluated using this sensor type. Some studies suggest that tyramine amounts can also be determined (Basozabal et al. 2014; Draz et al. 2021; Hidouri et al. 2021).

##### 4.3.3.2 Piezoelectric

The measurement method for piezoelectric sensors uses piezoelectric quartz crystals to record molecular interactions at their surfaces. Mass changes at the crystal surface occur due to analyte binding of biogenic amines, and a shift in the resonance frequency can be measured. These frequency shifts are proportional to the crystal's mass and the amount of biogenic amines measured.

This measurement is based on the quartz crystal microbalance (QCM). The alternating voltage from two electrodes is applied to the crystal, resulting in mechanical oscillation that, as described above, leads to a measurable resonance frequency in an oscillation circuit (Dai et al. 2014; Rocha‐Santos 2014; Pohanka 2018).

Another possible measurement setup is the so‐called surface acoustic wave (SAW), in which an alternating electric field is applied, resulting in vibrational or oscillatory motion and acoustic waves in the quartz crystal. The oscillator (i.e., the piezoelectric quartz crystal) vibrates at a characteristic frequency, and the shift in this frequency caused by analyte binding is detected, allowing quantification of the amount of biogenic amine (Pietrzyk et al. 2009; Y. Q. Fu et al. 2017).

This transducing method has been tested previously, primarily for detecting histamine, but studies on the quantification of cadaverine have also been conducted (Mutlu et al. 2008; Pietrzyk et al. 2009; Dai et al. 2014; Rocha‐Santos 2014; Y. Q. Fu et al. 2017).

##### 4.3.3.3 Optical

A biosensor with an optical transducing method consists of a biorecognition element or a biological sensing element connected to an optical transducer, which measures an optical signal resulting from, for example, absorption, transmission, luminescence, or reflectance when the analyte reacts with the electrode surface.

One possibility for an optical transducing system is surface plasmon resonance (SPR). It is based on the excitation of collective electron oscillations at the interface between a metal and a dielectric medium. It is characterized by its extremely high sensitivity to minute changes in refractive index at the nanometer scale (Jain et al. 2008; Basozabal et al. 2014; S. Jiang et al. 2015). In contrast, localized SPR describes the resonance phenomenon in which incident light excites free electrons in individual metal nanoparticles, typically gold or silver, to oscillate in unison. The resulting resonance wavelength and plasmonic signal intensity are closely linked to parameters such as particle size, shape, spacing, and the dielectric properties of the surrounding matrix. These can be used to detect biogenic amines in combination with MIP or specific antibodies, and detection limits in the microgram range can be achieved (Unser et al. 2015; Z. Zhang et al. 2018; Sharma et al. 2024; W. Zhang et al. 2023).

Another possibility is SERS, a label‐free, rapid, nondestructive method for detecting biogenic amines. This molecular vibration spectroscopy results from an inelastic scattering process of the molecules of interest. It is often used in combination with MIP to enhance the effectiveness. The limits of detection for biogenic amines range from 0 to 400 mg (Gao et al. 2015; Bingquan et al. 2017; Janči et al. 2017; Xie et al. 2017; Duan et al. 2023).

A relatively new approach for detecting biogenic amines is fiber‐optic sensors (FOBS). Optic fibers consist of a cylindrical core surrounded by a cladding. Due to the limited sensitivity and selectivity of optical fiber materials, they are often doped with germanium to increase the refractive index and enable total internal reflection. The detection of analytes, that is, biogenic amines, involves measuring changes in refractive index as concentrations vary. The limit of detection for biogenic amines is in the micrometer range (Pospiskova et al. 2013; Usman et al. 2016; W. Zhang et al. 2023).

### 4.4 Immunoassays

#### 4.4.1 Enzyme‐Linked Immunosorbent Assay (ELISA)

ELISA is a quantitative method that can also be used to determine the concentration of biogenic amines in food. Based on specific antigen–antibody interactions, it involves an enzymatically labeled antibody that produces a color reaction when a substrate is added, whose intensity is directly proportional to the amine concentration. Validation studies on fish samples, particularly those involving histamine, demonstrated high sensitivity and specificity, as well as strong correlations with HPLC analyses. ELISA scores highly in ease of use, low cost, high sample throughput, and automatability. However, limitations arise from possible cross‐reactions with matrix components and matrix effects, which can affect the accuracy of the results. Therefore, careful calibration in the respective sample is essential (Givanoudi et al. 2023; Shimoji et al. 2019).

## 5 Comparison and Evaluation of the Detection Methods

Method performance for biogenic amine detection in fish strongly depends on the chosen analytical approach, with histamine being the most commonly detected biogenic amine. As a result, comparing all possible method variations is rather difficult. Therefore, this review provides an overview of selected detection methods for biogenic amines and their specific detection limits in fish (Table 2). For further information, refer to the existing literature on comparing different detection methods, such as Papageorgiou et al. (2018), Önal et al. (2013), or Tırıs et al. (2023).

**TABLE 2:** Comparison of detection methods and limit of detection for biogenic amines in fish.

Method type	Detection method	Target amines	Derivatization	Limit of detection	Sample type	Reference
HPLC, IC–MS/MS	Ion chromatography tandem mass spectrometry (IC–MS/MS), high‐performance liquid chromatography (HPLC)	Cad, His, Phe, Put, Sperd, Sperm, Try, Tyr	Dansyl‐chloride (HPLC)	0.2–1.5 mg/kg (HPLC)	Anchovy (fresh, brined, dry‐salted, marinated, paste, smoked), Atlantic bonito, Herring (dry‐salted, marinated, fermented), salmon (paste, smoked), sardine (canned), tuna (paste, smoked)	Kočar et al. 2021
HPLC–UV/Vis	HPLC with UV/Vis detection	Cad, His, Phe, Put, Try	Dansyl‐chloride	0.14–0.5 mg/L	Golden dorado, Atlantic mackerel, amberjack	Pinto et al. 2016
HPLC–UV/FLD	HPLC with ultraviolet (UV) and fluorescence (FLD) detection	Cad, His, Put, Sperd, Sperm	Pyrene sulfonyl chloride	0.3–1.4 mg/kg	Sea bass, anchovy (fresh, marinated), mackerel (smoked), sardines (in oil), tuna (in water and salt)	Plakidi et al. 2020
HPLC–MS/MS	Liquid chromatography (HPLC) with tandem mass spectrometry (MS/MS)	Cad, His, Phe, Put, Sperd, Sperm, Try, Tyr	—	0.02–0.25 mg/kg	Small hake	Sagratini et al. 2012
CE–UV/Vis, HPLC–UV/Vis	CE and HPLC with UV/Vis detection	His	—	0.5 mg/kg (CE), 1 mg/kg (HPLC)	Tuna (in oil)	Cinquina et al. 2004
GC–FID	Gas chromatography (GC) with flame ionization detector (FID)	Cad, Hep, His, Sperd, Tyr	BSA + TMCS	1.2–2.9 mg/L	Fresh and salted fish: goldstripe sardine, Indo‐Pacific king mackerel, snakeskin gourami, whiptail stingray, toli shad	Munir et al. 2017
Raman spectroscopy	Surface‐enhanced Raman spectroscopy (SERS) with Au/Ag colloids	His	—	5 mg/kg	Mi‐iuy croaker	Bingquan et al. 2017
Enzyme biosensor	Enzymatic biosensor with monoamine oxidase, tyramine oxidase, diamine oxidase on screen‐printed thick‐film electrode (Ag/AgCl)	His, Put, Tyr	—	10 mg/kg (His, Tyr), 5 mg/kg (Put)	Cod, herring, salmon	Lange and Wittmann 2002
Enzyme biosensor	Enzymatic amperometric biosensor with monoamine oxidase (histidine decarboxylase, horseradish peroxidase) on carbon screen‐printed electrode	His	—	0.11 mg/L	Yellowfin tuna	Trevisani et al. 2019
Immunosensor	Immunosensor with monoclonal antibody and antibody‐based gold nanoparticle	His	—	0.01048 mg/kg (calculated), 0.25 mg/kg (visible)	Crucian carp, saury, tuna	Zeng et al. 2021
Immunosensor and aptasensor	Time‐gated Förster resonance energy transfer (TG‐FRET) immunosensor with monoclonal antibodies (IgG); aptasensor	His	—	0.19 mg/L (immunosensor), 0.03 mg/L (aptasensor)	Salmon	H.‐J. Fu et al. 2022
Aptasensor	Colorimetric aptasensor with HIS‐3 and TRY‐2 aptamers by systematic evolution of ligands by exponential enrichment (SELEX)	His, Try	—	0.001 mg/L (histamine), 0.01 mg/L (tryptamine)	Fish samples (not specified)	Duan et al. 2022
MIP sensor	Magnetic MIP electrochemical sensor	His	—	1.6 × 10^−6^ mg/L	Tuna	Hassan et al. 2019
MIP sensor	Biosensor with MIP on screen‐printed polyurethane electrode (LiClO4)	His	—	1.765 nM	Mackerel	Munir, Rahmawati, et al. 2023
Immunoassay	Biomimetic enzyme‐linked Immunoassay (BELISA) with MIP antibody	His, Try	—	0.04 mg/L (his), 0.14 mg/L (try)	Fish samples (not specified)	Peng et al. 2022
Immunoassay	Food ELISA, HistaSure ELISA (Testkits)	His	—	0.75 mg/kg	Anchovy (fresh, brined, dry‐salted, marinated, paste, smoked), Atlantic bonito, herring (dry‐salted, marinated, fermented), salmon (paste, smoked), sardine (canned), tuna (paste, smoked)	Kočar et al. 2021

Abbreviations: Cad, cadaverine; Hep, heptylamine; His, histamine; Phe, phenylethylamine; Put, putrescine; Sperd, spermidine; Sperm, spermine; Try, tryptamine; Tyr, tyramine.

Various extensive derivatization strategies of samples using reagents such as OPA and benzoyl chloride, combined with liquid chromatography detection methods, including UV, fluorescence, and MS/MS, can achieve detection limits ranging from approximately 0.02 to 1.5 mg/kg (Kočar et al. 2021; Pinto et al. 2016; Plakidi et al. 2020; Sagratini et al. 2012; Cinquina et al. 2004).

GC yields markedly different detection limits for biogenic amines in fish, ranging from 1.2 to 2.9 mg/L when coupled with flame ionization detection, indicating a less sensitive detection method. Similar to liquid chromatographic methods, extensive sample derivatization, for example, with *N*,*O*‐bis(trimethylsilyl)acetamide (BSA) and trimethylchlorosilane (TMCS), is necessary (Munir et al. 2017; Munir et al. 2021).

CE can achieve detection limits comparable to those of liquid chromatographic methods, with a limit of detection of around 0.5 mg/kg for histamine. Unlike other chromatographic detection methods, no sample derivatization is required (Cinquina et al. 2004).

Regarding NMR and NIR approaches for detecting biogenic amines, no studies were found on the detection limits of these compounds in fish. Furthermore, NMR spectroscopy is a rarely used detection method for biogenic amines and is still under investigation for its practicality in fish samples. When using SERS with gold and silver colloids, a limit of detection for histamine of 5 mg/kg was achieved (Bingquan et al. 2017).

Studies almost exclusively focus on histamine using biosensor and immunoassay methods. Enzyme‐based biosensors offer rapid, portable analysis with detection limits ranging from 0.11 mg/L to 10 mg/kg. However, some methods require additional validation and are susceptible to interference from other biogenic amines regarding the selectivity of the used enzyme (Trevisani et al. 2019; Lange and Wittmann 2002). In comparison, colorimetric aptasensors can achieve detection limits as low as 0.001 mg/L in fish samples (Duan et al. 2022).

The quantitative ranges reported by MIP‐based sensors vary by sensor type and procedure. For instance, one study using a magnetic MIP reported a limit of detection of 1.6 × 10^−6^ mg/L (Hassan et al. 2019), while another study using an MIP on a screen‐printed polyurethane electrode described a limit of detection of 1.765 nM (Munir, Jamal, et al. 2023).

Immunoassay methods have reported limits of 0.19 mg/L with a time‐gated Förster resonance energy transfer (TG‐FRET) immunosensor (H.‐J. Fu et al. 2022) and 0.01048 mg/kg with a gold nanoparticle immunosensor (Zeng et al. 2021). By contrast, ELISA‐based methods yield less consistent quantitative data. One study reported a limit of detection of 0.75 mg/kg for histamine (Kočar et al. 2021), and another reported limits of detection of 0.04 mg/L for histamine and 0.14 mg/L for tyramine using an MIP antibody (Peng et al. 2022).

The units used across studies varied (nM, µM, mM, mg/L, etc.), and differences in sample preparation make direct comparisons difficult. Each report details unique sample preparation techniques, operational parameters, and validation metrics. Overall, the papers suggest that the choice of biosensor technology significantly affects the detection limit for biogenic amines in fish, with aptamer‐based sensors achieving the lowest reported numerical limits.

Various fish species have been examined in the past and present for their biogenic amine content. Table 3 provides an overview of the world's most commercially relevant marine and freshwater fish species that have been examined more recently for their biogenic amine content. Detailed information about the applied detection method can be found in the mentioned references.

**TABLE 3:** Most relevant commercial fish species researched for biogenic amine content.

Fish family	Genus	Subspecies	Trade name	Target amines	References
Clupeidae	*Clupea*	*harengus*	Herring	Ag, Cad, His, Put, Sperd, Sperm, Tyr	Fernández‐Salguero and Mackie 1987; Özogul et al. 2002; Prester 2011
Clupeidae	*Engraulis*	*encrasicolus*	European anchovy	Ag, Cad, Dop, His, Oca, Phe, Put, Ser, Sperd, Sperm, Try, Tyr	Veciana‐Nogues et al. 1996; Pons‐Sánchez‐Cascado et al. 2006; Rossano et al. 2006
Clupeidae	*Sardina*	*pilchardus*	European sardine	Ag, Cad, His, Phe, Put, Sperm, Sperd, Try, Tyr	Ababouch et al. 1991; Aubourg et al. 1998; Özogul et al. 2006; Visciano et al. 2007; Prester et al. 2009
Cyprinidae	*Cyprinus*	*carpio*	Common carp	Cad, His, Phe, Put, Sperd, Sperm, Try, Tyr	Křížek et al. 2011; Prester 2011; Apetrei and Apetrei 2016; J. Zhang et al. 2019
Gadidae	*Gadus*	*morhua*	Atlantic cod	Cad, His, Phe, Put, Sperd, Sperm, Try, Tyr	Hernández‐Herrero et al. 2002; Zhai et al. 2012; Pawul‐Gruba et al. 2014
Gadidae	*Melanogrammus*	*aeglefinus*	Haddock	Cad, His, Put, Sperd, Sperm, Try, Tyr	Fernández‐Salguero and Mackie 1987; Rawles et al. 1996; Pawul‐Gruba et al. 2014
Merlucciidae	*Merluccius*	*merluccius*	Mediterranean hake	Ag, Cad, His, Put, Sperm	Baixas‐Nogueras et al. 2001; Ruiz‐Capillas and Moral 2001; Baixas‐Nogueras et al. 2003; Prester et al. 2009; Prester 2011
Salmonidae	*Oncorhynchus*	*mykiss*	Rainbow trout	Ag, Cad, His, Phe, Put, Sperd, Sperm, Try, Tyr	Yamanaka et al. 1989; Rodríguez et al. 1999; Chytiri et al. 2004; Al Bulushi et al. 2009; Prester 2011
Salmonidae	*Salmo*	*salar*	Atlantic salmon	His, Put, Tyr	Emborg et al. 2002; Lange and Wittmann 2002; Pawul‐Gruba et al. 2014
Scombridae	*Katsuwonus*	*pelamis*	Skipjack tuna	Cad, His, Put, Sperm, Tyr	Rossi et al. 2002; Al Bulushi et al. 2009; Prester 2011
Scombridae	*Scomber*	*scombrus*	Atlantic mackerel	Ag, Cad, Dop, His, Nor, Phe, Put, Ser, Sperd, Sperm, Try, Tyr	Fernández‐Salguero and Mackie 1979; Mendes 1999; Prester et al. 2009; M.‐K. Kim et al. 2009
Scombridae	*Scomber*	*japonicus*	Chub mackerel	Cad, His, Put, Tyr	Wendakoon et al. 1990; Mendes 1999; Prester 2011
Scombridae	*Thunnus*	*alalunga*	Albacore tuna	Cad, His, Put, Sperd, Sperm, Try, Tyr	Rawles et al. 1996; Lopez‐Galvez et al. 1995; Visciano et al. 2012
Scombridae	*Thunnus*	*albacares*	Yellowfin tuna	Cad, His, Put, Tyr	Guizani et al. 2005; Emborg et al. 2005; Du et al. 2002; Prester 2011; Silbande et al. 2016; Trevisani et al. 2019

Abbreviations: Ag, agmatine; Cad, cadaverine; Dop, dopamine; His, histamine; Met, methylbutylamine; Nor, noradrenaline; Oca, octopamine; Phe, phenylethylamine; Put, putrescine; Ser, Serine; Sperd, spermidine; Sperm, spermine; Tyr, tyramine.

A detailed table of all marine and freshwater fish species already examined, including those from more local markets, detailing their biogenic amine content, is available in Table S1. Although this table does not claim to be complete.

## 6 Conclusion

Several methods are available for detecting biogenic amines in fish. Despite being time‐consuming and costly, chromatographic techniques, particularly HPLC coupled with MS or UV/Vis detection, remain the gold standard due to their high reliability, sensitivity, and reproducibility. Biosensors offer a fast, simple alternative that does not require sample preparation, and they allow in‐line measurements. However, limitations in selectivity and long‐term stability currently restrict their industrial application. Spectroscopic methods, such as Raman spectroscopy and NMR spectroscopy, demonstrate high sensitivity and specificity; however, complex food matrices necessitate extensive data analysis. Overall, chromatographic methods are the most accurate and recommended approach for quantitatively determining multiple biogenic amines.

Intelligent packaging is an emerging research field, with ongoing studies integrating biorecognition elements directly into food packaging to detect degradation products associated with spoilage.

## Conflicts of Interest

The author declares no conflicts of interest.

## Supporting information




**Supporting Information**: jfds71083‐sup‐0001‐TableS1.pdf
