# Supplementary material for: Monitoring Biogenic Amines: Comparative Assessment of Detection Methods for Key Market Marine and Freshwater Species
Source: J Food Sci. 2026 Apr 29;91:e71083. doi: 10.1111/1750-3841.71083 (PMC13127245; doi:10.1111/1750-3841.71083)
Supplement: Supplementary file 1 — Supporting Information: jfds71083‐sup‐0001‐TableS1.pdf [file JFDS-91-0-s001.pdf]

Supplementary Table 1.: Examined fish species worldwide, researched for biogenic amine content

Fish family	Genus	Subspecies	Trade name	Target amines	Ref.
Anguilidae	*Anguilla*	*anguilla*	Eupaean eel	Ag, Cad, His, Put, Sperd, Sperm, Tyr, Try	(Y. Özogul et al. 2006)
Belonidae	*Belone*	*belone belone*	Garfish	His	(Dalgaard et al. 2006; Prester 2011)
Carangidae	*Carangoides*	*armatus*	Longfin Trevally	His	(Shakila et al. 2003)
Carangidae	*Decapterus*	*maruadsi*	Blue scad	Cad, His, Phe, Put, Sperd, Sperm, Try, Tyr	(Hu et al. 2012; Visciano et al. 2012; Zhai et al. 2012)
Carangidae	*Selar*	*crumenophthalmus*	Bigeye scad	Cad, His, Phe, Put, Sperd, Sperm, Try, Tyr	(Zhai et al. 2012)
Carangidae	*Seriola*	*brama*	Blue warehou	His	(Pawul-Gruba et al. 2014)
Carangidae	*Seriola*	*dumerili*	Amber-jack	Cad, His, Phe, Put, Sperd, Sperm, Try, Tyr	(Kim et al. 2009; Visciano et al. 2012; Pinto et al. 2016)
Carangidae	*Seriola*	*grandis*	Kingfish	His	(Fletcher et al. 1995; Rawles et al. 1996)
Carangidae	*Seriola*	*lalandi*	Yellowtail amber-jack	His	(Auerswald et al. 2006; Prester 2011)
Carangidae	*Trachinotus*	*blochii*	Golden pompano	Cad, His, Phe, Put, Sperd, Sperm, Try, Tyr	(Visciano et al. 2012; Zhai et al. 2012)

Fish family	Genus	Subspecies	Trade name	Target amines	Ref.
Carangidae	*Trachurus*	*japonicus*	Japanese jack mackerel, Pacific mackerel	Cad, His, Put, Sperd, Sperm, Try, Tyr	(Kim et al. 2001; 2009; Visciano et al. 2012)
Carangidae	*Trachurus*	*trachurus*	Atlantic horse mackerel	Ag, Cad, His. Put	(Mendes 1999)
Cepolidae	*Cepola*	*spp.*	Bandfish	Cad, His, Phe, Put, Sperd, Sperm, Try, Tyr	(Visciano et al. 2012; Zhai et al. 2012)
Chanidae	*Chanos*	*chanos*	Milkfish	His	(Tsai et al. 2007; Prester 2011)
Chiasmodontidae	*Chiasmodon*	*niger*	Swallow fish	Cad, His, Phe, Put, Sperd, Sperm, Try, Tyr	(Zhai et al. 2012)
Clupeidae	*Clupea*	*harengus*	Herring	Ag, Cad, His, Put, Sperd, Sperm, Tyr	(Fernández-Salguero and Mackie 1987; Özogul et al. 2002; Prester 2011)
Clupeidae	*Clupea*	*pallasii*	Pacific herring	Cad, His, Put, Sperd, Sperm, Try, Tyr	(Kim et al. 2009; Visciano et al. 2012)
Clupeidae	*Engraulis*	*encrasicolus*	European anchovy	Ag, Cad, Dop, His, Oc, Phe, Put, Ser, Sperd, Sperm, Try, Tyr	(Veciana-Nogues et al. 1996; Pons-Sánchez-Cascado et al. 2006; Rossano et al. 2006)

Fish family	Genus	Subspecies	Trade name	Target amines	Ref.
Clupeidae	*Sardina*	*pilchardus*	European sardine	Ag, Cad, His, Phe, Put, Sperm, Sperd, Try, Tyr,	(Ababouch et al. 1991; Aubourg et al. 1998; Y. Özogul et al. 2006; Visciano et al. 2007; Prester et al. 2009)
Clupeidae	*Sardinella*	*aurita*	Atlantic sardine	Cad, His, Put	(Bomke et al. 2009)
Clupeidae	*Sardinella*	*fimbriata*	Fringe-scale sardinella	His	(Shakila et al. 2003)
Clupeidae	*Sardinella*	*gibbosa*	Gold-stripe sardinella	Cad, Hep, His, Sperm, Tyr	(Munir et al. 2017)
Clupeidae	*Sardinops*	*sagax*	Pacific sardine	Cad, His, Put	(Bomke et al. 2009)
Clupeidae	*Sprattus*	*sprattus*	European spratt	His	(Pawul-Gruba et al. 2014)
Clupeidae	*Tenualosa*	*toli*	Toli shad	Cad, Hep, His, Sperm, Tyr	(Munir et al. 2017)
Coryphaenidae	*Coryphaena*	*hippurus*	Mahi-Mahi	Cad, His, Put	(Al Bulushi et al. 2009; Prester 2011; Self and Wu 2012)
Cyprinidae	*Carassius*	*carassius*	Crucian carp	Cad, His, Phe, Put, Sperd, Sperm, Try, Tyr	(Zhai et al. 2012; Apetrei and Apetrei 2016; Zeng et al. 2021)
Cyprinidae	*Cirrhinus*	*molitorella*	Mud carp	Cad, His, Phe, Put, Sperd, Sperm, Try, Tyr	(Zhai et al. 2012)

Fish family	Genus	Subspecies	Trade name	Target amines	Ref.
Cyprinidae	*Ctenopharyngodon*	*idella*	Grass carp	Ag, Cad, Dop, His, Phe, Put, Ser, Sperd, Sperm, Try, Tyr	(Moon et al. 2010; Zhai et al. 2012; Zhang et al. 2019)
Cyprinidae	*Cyprinus*	*carpio*	Common carp	Cad, His, Phe, Put, Sperd, Sperm, Try, Tyr	(Křížek et al. 2011; Prester 2011; Apetrei and Apetrei 2016; Zhang et al. 2019)
Cyprinidae	*Hypophthalmichthys*	*molixtrix*	Silver carp	Phe, Put, Try, Tyr	(Bóka et al. 2012)
Cyprinidae	*Rutilus*	*rutilus*	Rudd, Roach	His	(Pawul-Gruba et al. 2014)
Engraulidae	*Stolephorus*	*indicus*	Indian anchovy	Cad, His, Put, Sperd, Sperm, Try, Tyr	(Yongsawatdigul et al. 2004; Visciano et al. 2012)
Ephippidae	*Ephippus*	*orbis*	Orbfish	Cad, His, Phe, Put, Sperd, Sperm, Try, Tyr	(Zhai et al. 2012)
Esocidae	*Esox*	*lucius*	Northern pike	Cad, His, Phe, Put, Try, Tyr, Sperd, sperm	(Pawul-Gruba et al. 2014; Shang et al. 2023)
Gadidae	*Gadus*	*chalcogrammus*	Alaska pollock	Cad, His, Put, Sperd, Sperm, Try, Tyr	(Kim et al. 2009; Visciano et al. 2012)
Gadidae	*Gadus*	*macrocephalus*	Pacific cod	Cad, His, Phe, Put, Sperd, Sperm, Try, Tyr	(Kim et al. 2009; Visciano et al. 2012; Ishimaru et al. 2019)

Fish family	Genus	Subspecies	Trade name	Target amines	Ref.
Gadidae	*Gadus*	*morhua*	Atlantic cod	Cad, His, Phe, Put, Sperd, Sperm, Try, Tyr	(Hernández-Herrero et al. 2002; Zhai et al. 2012; Pawul-Gruba et al. 2014)
Gadidae	*Melanogrammus*	*aeglefinus*	Haddock	Cad, His, Put, Sperd, Sperm, Try, Tyr	(Fernández-Salguero and Mackie 1987; Rawles et al. 1996; Pawul-Gruba et al. 2014)
Gadidae	*Merlangius*	*merlangus*	Whiting	Cad, His, Met, Put, Sperd, Sperm, Try, Tyr	(Duflos et al. 1999; Al Bulushi et al. 2009)
Gadidae	*Pollachius*	*virens*	Pollock	His	(Pawul-Gruba et al. 2014)
Gempylidae	*Lepidocybium*	*flavobrunneum*	Escolar	His	(Feldman et al. 2005; Prester 2011)
Istiophoridae	*Istiophorus*	*spp.*	Sailfish	Cad, His, Put, Sperd, Sperm, Try, Tyr	(Tsai et al. 2004; Visciano et al. 2012)
Istiophoridae	*Makaira*	*nigricans*	Atlantic blue marlin	His	(Tsai et al. 2007; Prester 2011))
Istiophoridae	*Tetrapturus*	*angustirostris*	Shortbill spearfish	Ag, Cad, His, Phe, Put, Sperd, Sperm	(Chen et al. 2010; Prester 2011)
Latidae	*Lates*	*calcarifer*	Barra-mundi	Ag, Cad, His, Phe, Put, Sperd, Sperm, Try, Tyr	(Yassoralipour et al. 2016)

Fish family	Genus	Subspecies	Trade name	Target amines	Ref.
Lethrinidae	*Lethrinus*	*miniatus*	Emperor bream	His	(Shakila et al. 2003; Prester 2011)
Leuciscidae	*Abramis*	*brama*	Bream	His	(Pawul-Gruba et al. 2014)
Macrourinae	*Coryphaenoides*	*rupestris*	Grenadier	His	(Pawul-Gruba et al. 2014)
Merlucciidae	*Macruronus*	*magellanicus*	Hoki	His	(Pawul-Gruba et al. 2014)
Merlucciidae	*Merluccius*	*carpensis*	Hake	His	(Auerswald et al. 2006; Prester 2011)
Merlucciidae	*Merluccius*	*merluccius*	Medi-terranean hake	Ag, Cad, His, Put, Sperm	(Baixas-Nogueras et al. 2001; Ruiz-Capillas and Moral 2001; Baixas-Nogueras et al. 2003; Prester et al. 2009; Prester 2011)
Merlucciidae	*Merluccius*	*productus*	Pacific Hake, Small hake	Cad, His, Phe, Put, Sperd, Sperm, Try, Tyr	(Sagratini et al. 2012)
Moronidae	*Dicentrarchus*	*labrax*	Medi-terranean sea bass	Cad, Put, Sperd, Sperm, Try, Tyr	(Paleologos et al. 2004; Al Bulushi et al. 2009; Kim et al. 2009)
Mugilidae	*Mugil*	*cephalus*	Flathead grey mullet	Cad, His, Phe, Put, Sperd, Sperm, Try, Tyr	(Zhai et al. 2012)

Fish family	Genus	Subspecies	Trade name	Target amines	Ref.
Nemipteridae	*Nemipterus*	*gracilis*	Graceful threadfin bream	Cad, His, Phe, Put, Sperd, Sperm, Try, Tyr	(Zhai et al. 2012)
Nemipteridae	*Nemipterus*	*virgatus*	golden thread	Cad, His, Put, Sperd, Sperm, Try, Tyr	(Hu et al. 2012)
Odontobutidae	*Perccottus*	*glenii*	Chinese sleeper	Cad, His, Phe, Put, Sperd, Sperm, Try, Tyr	(Zhai et al. 2012)
Osmeridae	*Mallotus*	*villosus*	Capelin	His	(Aksnes and Brekken 1988; Rawles et al. 1996)
Osphronemidae	*Trichopodus*	*pectoralis*	Snakeskin gourami	Cad, Hep, His, Sperm, Tyr	(Munir et al. 2017)
Pangasiidae	*Pangasius*	*bocourti*	Basa	Cad, His, Iso, Phe, Put, Sperd, Sperm, Try, Tyr	(Kaufmann and Maden 2018)
Pangasiidae	*Pangasius*	*hypopthalmus*	Striped catfish	Cad, His, Iso, Phe, Put, Sperd, Sperm, Try, Tyr	(Kaufmann and Maden 2018)
Percidae	*Perca*	*fluviatilis*	European perch	Cad, His, Put, Sperd, Sperm, Try, Tyr	(Křížek et al. 2011; Apetrei and Apetrei 2016)
Percidae	*Sander*	*lucioperca*	Zander, Pike perch	Cad, His, Put, Tyr	(Ehsani and Jasour 2012)

Fish family	Genus	Subspecies	Trade name	Target amines	Ref.
Percomorphaceae	*Arripis*	*trutta*	Australian salmon	His	(Fletcher et al. 1995; Rawles et al. 1996)
Pholidae	*Pholis*	*gunellus*	Rock gunnel	Cad, His, Phe, Put, Sperd, Sperm, Try, Tyr	(Bomke et al. 2009; Zhai et al. 2012)
Pleuronectidae	*Platichthys*	*flesus*	Flounder	His	(Pawul-Gruba et al. 2014)
Pleuronectidae	*Pleuronectes*	*platessa*	Seeplaice	Cad, His, Met, Put, Sperd, Sperm, Try, Tyr	(Duflos et al. 1999; Al Bulushi et al. 2009; Tsoukalas et al. 2022)
Salmininae	*Salminus*	*brasiliensis*	Golden dorado	Cad, His, Phe, Put, Try	(Pinto et al. 2016)
Salmonidae	*Oncorhynchus*	*keta*	Chum salmon	Cad, His, Put, Sperd, Sperm, Try, Tyr	(Yamanaka et al. 1989; Guo et al. 2022)
Salmonidae	*Oncorhynchus*	*kisutch*	Coho salmon	His	(Aubourg et al. 2007)
Salmonidae	*Oncorhynchus*	*gorbuscha*	Pink salmon	His	(Crapo and Himelbloom 1999)
Salmonidae	*Oncorhynchus*	*mykiss*	Rainbow trout	Ag, Cad, His, Phe, Put, Sperd, Sperm, Try, Tyr	(Yamanaka et al. 1989; Rodrguez et al. 1999; Chytiri et al. 2004; Al Bulushi et al. 2009; Prester 2011)
Salmonidae	*Oncorhynchus*	*nerka*	Sockeye salmon	Ag, Cad, His, Phe, Put, Try, Tyr	(Self and Wu 2012)

Fish family	Genus	Subspecies	Trade name	Target amines	Ref.
Salmonidae	*Salmo*	*salar*	Atlantic salmon	His, Put, Tyr	(Emborg et al. 2002; Lange and Wittmann 2002; Pawul-Gruba et al. 2014)
Salmonidae	*Salmo*	*trutta*	Brown trout	Ag, Cad, Coa, Dia, Eta, His, Met, Oca, Phe, Put, Pyr, Sperd, Sperm, Try, Tyr	(Pawul-Gruba et al. 2014; Kosma and Badeka 2021; Moser et al. 2023)
Sciaenidae	*Larimichthys*	*crocea*	Large yellow croaker	Cad, His, Put, Sperd, Sperm, Try, Tyr	(Hu et al. 2012; Zhang et al. 2021)
Sciaenidae	*Larimichthys*	*polyactis*	Yellow croaker	Cad, His, Phe, Put, Try, Tyr	(Zhang et al. 2019)
Sciaenidae	*Miichthys*	*miiuy*	Mi-uiy croaker, Brown croaker	His	(Bingquan et al. 2017)
Scomberesocidae	*Cololabis*	*saira*	Pacific saury	Cad, His, Phe, Put, Sperd, Sperm, Try, Tyr	(Kim et al. 2009; Hu et al. 2012; Visciano et al. 2012; Zhai et al. 2012)
Scombridae	*Katsuwonus*	*pelamis*	Skipjack tuna	Cad, His, Put, Sperm, Tyr	(Rossi et al. 2002; Al Bulushi et al. 2009; Munir et al. 2023)
Scombridae	*Rastrellinger*	*kanagurta*	Indian mackerel	His	(Shakila et al. 2003; Chong et al. 2014; Munir et al. 2023)

Fish family	Genus	Subspecies	Trade name	Target amines	Ref.
Scombridae	*Sarda*	*sarda*	Atlantic bonito	His	(Kočar et al. 2021)
Scombridae	*Scomber*	*japonicus*	Chub mackerel	Ag, Cad, His, Put, Tyr	(Wendakoon et al. 1990; Mendes 1999; Prester 2011; Jiang et al. 2013)
Scombridae	*Scomber*	*scombrus*	Atlantic mackerel	Ag, Cad, Dop, His, Nor, Phe, Put, Ser, Sperd, Sperm, Try, Tyr	(Fernández-Salguero and Mackie 1979; Mendes 1999; Kim et al. 2009; Prester et al. 2009)
Scombridae	*Scomberomorus*	*guttatus*	Indo-Pacific king mackerel	Cad, Hep, His, Sperm, Tyr	(Munir et al. 2017)
Scombridae	*Scomberomorus*	*maculatus*	Spanish mackerel	Cad, His, Phe, Put, Sperd, Sperm, Try, Tyr	(Middlebrooks et al. 1988; Zhai et al. 2012)
Scombridae	*Scomberomorus*	*niphonius*	Japanese spanish mackerel	Cad, His, Phe, Put, Try, Tyr	(Zhang et al. 2019)
Scombridae	*Thunnus*	*alalunga*	Albacore tuna	Cad, His, Put, Sperd, Sperm, Try, Tyr	(Lopez-Galvez et al. 1995; Rawles et al. 1996; Visciano et al. 2012)

Fish family	Genus	Subspecies	Trade name	Target amines	Ref.
Scombridae	*Thunnus*	*albacares*	Yellowfin tuna	Cad, His, Put, Tyr	(Du et al. 2002; Emborg et al. 2005; Guizani et al. 2005; Prester 2011; Silbande et al. 2016; Trevisani et al. 2019)
Scombridae	*Thunnus*	*obesus*	Bigeye tuna	Cad, His, Put	(Rossi et al. 2002; Prester 2011)
Scombridae	*Thunnus*	*thynnus*	Atlantic bluefin tuna	Cad, His, Phe, Put, Tyr	(Veciana-Nogués et al. 1997)
Scombridae	*Thunnus*	*tonggol*	Longtail tuna	Cad, His, Put, Sperm	(Bita and Sharifian 2024)
Scophthalmidae	*Scophthalmus*	*maximus*	Turbot	Cad, His, Phe, Put, Sperd, Sperm, Tma, Try, Tyr	(Yesim Özogul et al. 2006)
Sebastidae	*Sebastes*	*alutus*	Red perch	His	(Feier and Goetsch 1993; Rawles et al. 1996)
Sebastidae	*Sebastes*	*marinus*	Redfish	His	(Pawul-Gruba et al. 2014)
Sebastidae	*Sebastes*	*spp.*	Rockfish	Cad, His, Put, Sperd, Sperm	(Mietz and Karmas 1978; Al Bulushi et al. 2009)
Serranidae	*Centropristis*	*striata*	Black sea bass	Cad, His, Put, Sperd, Sperm	(Plakidi et al. 2020)
Siluridae	*Silurus*	*glanis*	European catfish, Wels catfish	His	(Apetrei and Apetrei 2016)

Fish family	Genus	Subspecies	Trade name	Target amines	Ref.
Sinipercidae	*Siniperca*	*chuatsi*	Chinese perch	Cad, His, Phe, Put, Sperd, Sperm, Try, Tyr	(Zhai et al. 2012)
Sparidae	*Dentex*	*tumifroms*	Yellow seabream	Cad, His, Put, Sperd, Sperm, Try, Tyr	(Hu et al. 2012)
Sparidae	*Sparus*	*aurata*	Gilthead seabream	Tma	(Lougovois et al. 2003)
Sphyraenidae	*Sphyraena*	*barracuda*	Great barracuda	His	(Shakila et al. 2003; Prester 2011)
Stromateidae	*Pampus*	*argenteus*	Silver pomfret	Cad, His, Put, Sperd, Sperm, Try, Tyr	(Hu et al. 2012; Guo et al. 2022)
Tincidae	*Tinca*	*tinca*	Tench	His	(Apetrei and Apetrei 2016)
Trachinidae	*Trachinus*	*draco*	Greater weever	His	(Pérez et al. 2013)
Trichiurinae	*Trichiurus*	*haumela*	Belt fish	Cad, His, Phe, Put, Sperd, Sperm, Try, Tyr	(Hu et al. 2012; Zhai et al. 2012)
Trichiurinae	*Trichiurus*	*lepturus*	Large-head hairtail	Ag, Ben, Cad, Dia, Dop, His, Oca, Phe, Put, Ser, Sperd, Sperm, Try, Tyr	(Hu et al. 2012; Deng et al. 2024)
Xenocrypididae	*Megalobrama*	*amblycephala*	Wuchang bream	Cad, His, Phe, Put, Sperd, Sperm, Try, Tyr	(Zhai et al. 2012)

**Fish family**	**Genus**	**Subspecies**	**Trade name**	**Target amines**	**Ref.**
Xiphiidae	*Xiphias*	*gladius*	Broadbill swordfish	His	(Chang et al. 2008; Prester 2011)
